# Analysis of Rydberg Series of Ytterbium Monofluoride, YbF

**DOI:** 10.1021/acs.jpca.5c03336

**Published:** 2025-06-17

**Authors:** Luca Diaconescu, Sascha Schaller, André Fielicke, Gerard Meijer

**Affiliations:** 28259 Fritz-Haber-Institut der Max-Planck-Gesellschaft, Faradayweg 4-6, Berlin 14195, Germany

## Abstract

Resonance-enhanced multiphoton ionization (REMPI) is used to characterize Rydberg states of ^174^YbF. Assignment of these Rydberg states to series that converge to various rotational and vibrational levels of the ^174^YbF^+^ cation leads to an accurate value for the ionization energy *IE* = 48706.57(8) cm^–1^ of YbF. The values for the rotational constants of the YbF^+^ X^1^Σ^+^ ground state are found as *B*_ν = 0_^+^ = 0.257(1) cm^–1^ and *B*_ν = 1_^+^ = 0.255(3) cm^–1^, while the vibrational transition energy is Δ*G*_1/2_ = 598.86(15) cm^–1^. Photoinduced Rydberg ionization (PIRI) measurements via a high-lying Rydberg state have been performed using an infrared free electron laser, confirming the Δ*G*_1/2_ value.

## Graphical abstract

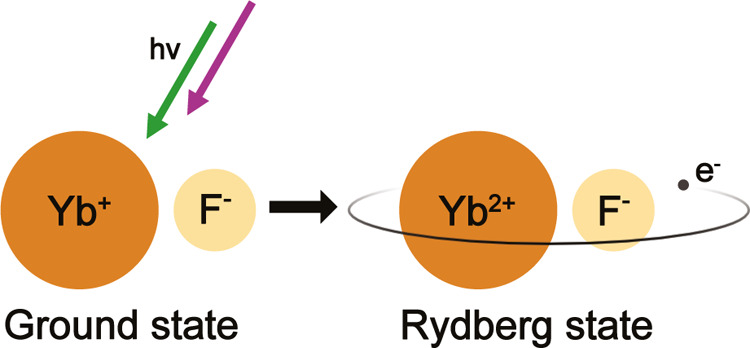

## Introduction

Atomic and molecular Rydberg states, where a single electron is highly excited, possess several “extreme” properties that scale strongly with the principal quantum number *n*, such as large Rydberg electron orbits, large polarizabilities and long lifetimes^1^. These systems are very interesting from a fundamental physical and chemical point of view, as well as due to the potential role they play in natural processes such as the dissociative recombination of ions in diffuse interstellar clouds and in the ionosphere^2–4^ . They also open the door for applications in the realms of quantum information processing^5,6^ and quantum simulation^7,8^ . Furthermore, Rydberg states enable the acquisition of valuable spectroscopic data, as they form series that converge to energy levels of the cationic core of the respective atom or molecule. Such studies have been demonstrated on numerous systems^9–12^ . Spectroscopic techniques that rely on Rydberg states specifically have also been developed, most notably the zero electron kinetic energy (ZEKE)^13–16^ , the mass-analyzed threshold ionization (MATI)^17–19^ , and the photoinduced Rydberg ionization (PIRI)^20–22^ methods.

The ytterbium monofluoride (YbF) molecule has attracted attention as a suitable platform for experiments to determine the electric dipole moment of the electron (eEDM)^23–25^ . Several of its electronic states with term energies below 28,000 cm^–1^ had already been spectroscopically observed and characterized by the turn of the 21st century.^26–29^ Significant effort has also gone into the study of fine and hyperfine interactions in the ground and first excited state^30–32^ of YbF, and of vibrational branching ratios relevant to laser cooling^33,34^ . More recently, the low-lying electronic states of YbF with an inner 4f-shell excitation, i.e., with a “4f hole” configuration of Yb^+^(4f^13^6s^2^)­F^–^ (as opposed to the Yb^+^(4f^14^6s^1^)­F^–^ “nonhole” configuration of the X^2^Σ^+^ ground state), have been studied in detail,^35–37^ along with electronic states of hybrid (i.e., hole and nonhole) character.^37,38^ While such 4f to 6s excitations are specific for lanthanide atoms like Yb, they have not been as thoroughly studied in lanthanide containing molecules.

Here, mass-resolved spectroscopy of high-lying Rydberg states (effective principal quantum number ≥ 20) of the ^174^YbF molecule is presented, thereby providing accurate information on the ionization energy of YbF and on the rotational and vibrational constants of YbF^+^. UV–vis resonance-enhanced multiphoton ionization (REMPI) via an intermediate electronically excited state has been employed to obtain rotationally resolved spectra of high-lying Rydberg states. The study is focused on the regions where states of zero or one quanta of vibrational excitation are expected.

Using the PIRI technique, where a high-lying Rydberg state is first prepared and then ionized by infrared (IR) irradiation of the cationic core, the vibrational information on the YbF^+^ ground state obtained from the analysis of the REMPI spectra has been confirmed. The IR-PIRI technique, while having been used for characterizing vibrational modes of larger organic molecules^39–43^ , had thus far found very little application to diatomic molecules.

## Experimental Methods

All measurements are performed using the laser ablation/molecular beam spectroscopy setup^44^ shown in Figure 1(a), operating at 10 Hz. The ytterbium monofluoride molecules are produced by laser ablation^45^ of a rotating-translating ytterbium rod and sulfur hexafluoride (SF_6_) pulsed into the source in 0.1% concentration in He carrier gas at a backing pressure of 4 bar. The hot molecules then undergo cooling by supersonic expansion down to a rotational temperature of ≈ 40 K. The molecular beam is subsequently trimmed by a skimmer and passes between two deflection plates that remove most of the ionic species produced in the ablation process.

**Figure 1:**
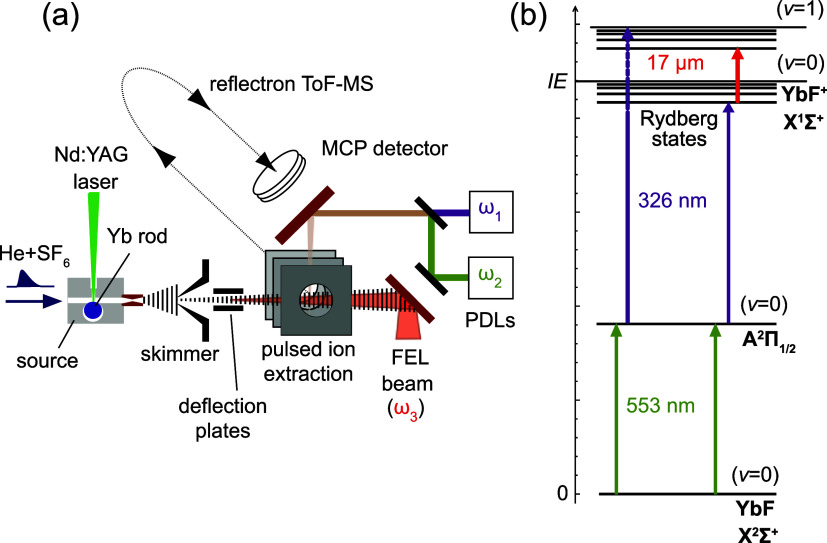
(a) Setup used for REMPI, PIRI and MATI scans on molecular beams. A molecular beam of YbF seeded in He is produced by laser ablation followed by supersonic expansion, and interacts with light from up to three wavelength-tunable lasers; (b) Schematic of UV–vis REMPI (excitation with visible radiation into a well-defined intermediate rotational state, followed by a UV scan) and PIRI (preparation of a nonautoionizing Rydberg state, and subsequent IR scan) methods used in this work.

The neutral YbF molecules then interact with light from two pulsed dye lasers (PDLs; Sirah PrecisionScan, 0.04 cm^–1^ line width; Radiant Dyes NarrowScan, 0.05 cm^–1^ line width) that is perpendicularly coupled into the extraction region. Their wavelength is measured by either a HighFinesse WS6–600 wavemeter or a MOGLabs FZW600 wavemeter, both having a nominal absolute accuracy of 0.02 cm^–1^. The ions generated by the incident light are then perpendicularly extracted into a reflectron time-of-flight mass spectrometer (R. M. Jordan Inc.) by applying a pulsed voltage to the extraction plates and are ultimately detected by an MCP detector. All stable isotopologues of ^*x*^Yb^19^F (*x* = 168, 170–174, 176) are present in the molecular beam. The isotopologue of highest abundance, ^174^YbF, is the one being used in eEDM experiments and has hence been selected as main focus here. It conveniently offers high signal along with spectral simplicity due to the lack of nuclear spin of ^174^Yb. For PIRI scans, we make use of the in-house infrared free electron laser (FHI-FEL^46^) in addition to the two PDLs described above. The FEL radiation, with a bandwidth of about 0.3% of the central wavelength, counter-propagates the molecular beam. For the sake of better overlap, the IR beam is out of focus in the extraction region, resulting in a beamwidth of around 3 mm. The individual FEL macropulses come at a repetition rate of 10 Hz, have a length of ≈ 10 μs, and are comprised of (subpicosecond) micropulses separated by 1 ns each. The macropulse energy reaches 40 mJ and can be controlled with a series of attenuators.

The two types of measurements performed in this study, (1 + 1′)-REMPI and IR-PIRI, are schematically shown in Figure 1(b). For REMPI, the well-known A^2^Π_1/2_ ← X^2^Σ^+^ transitions of YbF are used to selectively populate a rotational level of the A^2^Π_1/2_ excited state. Then, after a brief delay of ≈ 10 ns, the light of a second laser interacts with the molecules, driving transitions from the intermediate level into high-lying Rydberg states. This delay between the lasers, while being short enough as to not lead to significant population decay from the intermediate A^2^Π_1/2_ state (τ = 24(1) ns, ref. 37), ensures that the desired excitation scheme is achieved. The Rydberg states with energies above the ionization energy (*IE*) autoionize, while the ones with energies below the (field-free) *IE* can only autoionize when the extraction *E*-field is applied.^47^ To ensure that potential (short-lived) high-lying non-Rydberg states do not get field-ionized along with the (long-lived) Rydberg states, i.e., to obtain a clean spectrum of the Rydberg series, the extraction is delayed by about 2 μs. Scans via more than a dozen intermediate rotational levels of both parities are performed, with 30 mass spectra averaged per wavelength point, step sizes of either 0.06 or 0.12 cm^–1^, excitation laser fluence ranging between 1 and 70 μJ/cm^2^ and ionization laser fluence ranging between 75 and 100 μJ/cm^2^.

For PIRI, a (nonvibrationally excited) Rydberg state identified by REMPI is selectively prepared using two lasers. Infrared radiation then drives the transition from this Rydberg state to a vibrationally excited one. The final state autoionizes and is then mass-spectrometrically detected. Since it is assumed that the loosely bound Rydberg electron barely interacts with the cationic core, spectra obtained by this method closely resemble those of the bare cation. To counteract short-term fluctuations related to the laser ablation source, and to improve the quality of the PIRI spectra, we employ the MATI technique: A small DC bias voltage is applied (here 6.2 V) to the extraction plate of the mass spectrometer, in order to spatially separate the photoinduced ions from the neutral species. Upon applying the extraction field with a delay of ≈ 2 μs, the neutral Rydberg molecules autoionize and are then extracted from a different location than the photoions, leading to temporal separation in the ToF mass spectrum. The sum of photo and field-induced signals is indicative of the total number of molecules prepared in the intermediate Rydberg state, and is used for normalization.

The PIRI scans are performed via a single intermediate Rydberg state (*N* ^+^ = 4, n* = 36.12), at 300 mass spectra averaged per wavelength point, a step size of 1 cm^–1^, and 10 mJ macropulse energy for the IR-FEL.

## Results and Discussion

### Rydberg Series

Figure 2 shows REMPI spectra obtained via three rotational states of the intermediate A^2^Π_1/2_ state, along with the main Rydberg series found therein. Being in the energy region of the ionization energy (known to be around 48703(5) cm^–1^, ref 36.), these Rydberg series are not vibrationally excited. As described in more detail below, the series shown in Figure 2(a) is found to converge to the lowest rotational state of YbF^+^ (i.e., *N* ^+^ = 0) and thus its limit *E*_lim_ = 48706.68(21) cm^–1^ corresponds to the *IE* of YbF. Further below, this value will be refined by taking into account the limits of additional rotationally excited series. The general signal increase that is visible in all three spectra beyond 48,700 cm^–1^ is due to excitation into the ionization continuum, as opposed to the lower energy spectral regions, where ionization is facilitated by the pulsed extraction field.

**Figure 2:**
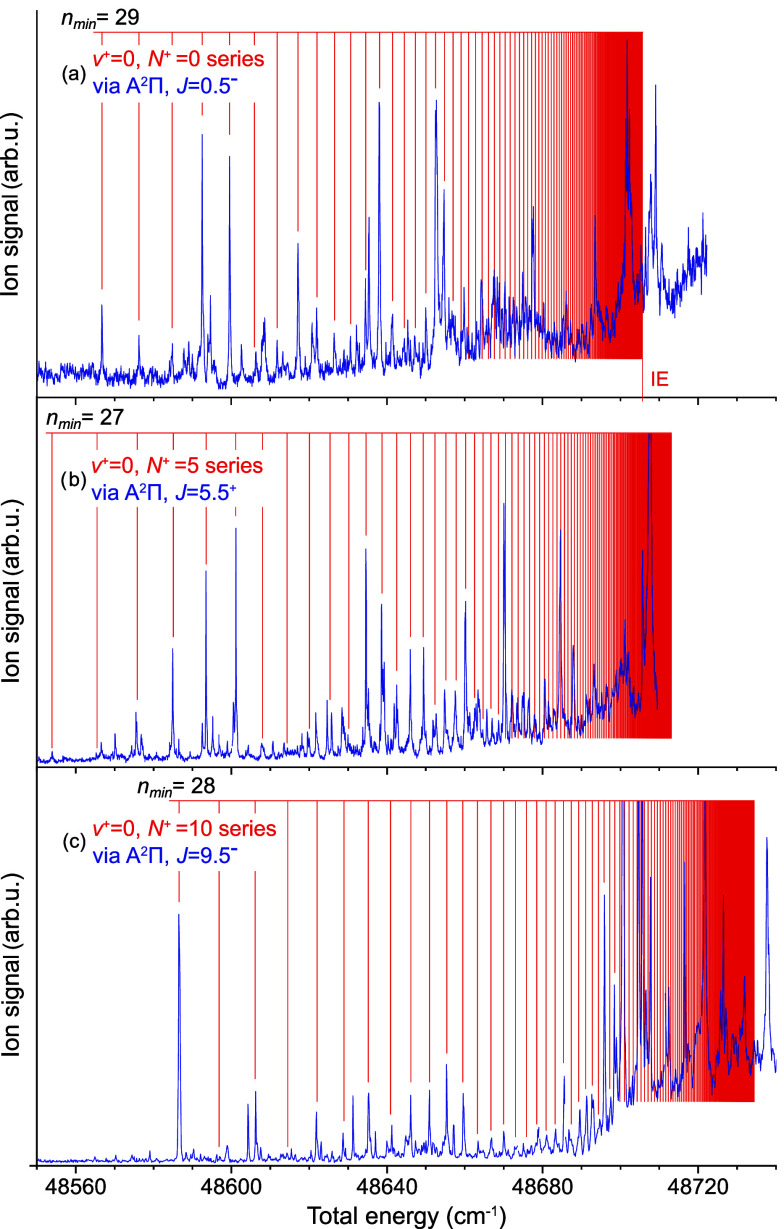
(1 + 1′)-REMPI spectra measured via three different rotational levels of A^2^Π_1/2_ along with the identified ν = 0 Rydberg series. The limit of the lowest-lying Rydberg series (a) corresponds to the *IE* of YbF. The (lowest possible) *n* quantum number of a low-lying series member is given in the upper left corner of each spectrum.

All the recorded spectra show one dominant Rydberg series. In some cases “secondary” series are also observed, see for example the numerous peaks in Figure 2(b) that do not belong to the marked *N* ^+^ = 5 series. We have chosen to restrict our analysis to the dominant series in each case, as it is easiest identified and leads to the most accurate values for its limit, needed for the subsequent determination of spectroscopic constants. Furthermore, the dominant series observed depends on the total angular momentum (excluding nuclear spin) quantum number *J* of the intermediate state. Due to transitioning between Hund’s case (a) in the A^2^Π_1/2_ state and Hund’s case (d) in the high-lying Rydberg states, the rotational and angular momentum selection rules are nontrivial and weakened, meaning that a large number of *N* ^+^ are potentially accessible from a given intermediate *J*.^48^ Still, the intuitive propensity rule *N* ^+^ ≈ *J* has been empirically observed before^49^ and is confirmed by our measurement. Analogously, Rydberg series of various *l* should be reachable. By comparing the spin orbit splitting of the A^2^Π intermediate state to that of known states of the Yb^+^ cation, the A^2^Π state is concluded to have dominant d (i.e., *l* = 2) character. Therefore, Rydberg states of *l* = 1, 3 are easiest accessible, in accordance with the hydrogenic angular momentum selection rule Δ*l* = ±1. For a thorough understanding of all of the observed transitions in the eight measured spectra, a multichannel quantum defect theory analysis^50^ is necessary. This is however beyond the scope of the present study, that mostly aims at the extraction of spectroscopic data on YbF and YbF^+^. We assume that interseries interactions do not play a dominant role here, since characteristic spectral patterns hinting at perturbations are mostly missing, and since the dominant series in each spectrum could be fitted well over a significant *n* range, assuming a constant quantum defect (see below).

For a molecule the (unperturbed) energies of the Rydberg states making up a Rydberg series are given by

1 $$E_{n,l,N^{+},\nu^{+}}=\mathrm{IE}+E_{\mathrm{el}}^{+}+E_{\mathrm{vib}}^{+}\left(\nu^{+}\right)+E_{\mathrm{rot}}^{+}\left(N^{+}\right)-\frac{R_{\mu }}{{\left(n-\delta_{n,l}\right)}^{2}}$$

where *IE* is the ionization energy, *R*_μ_ is the mass corrected Rydberg constant, δ_*n*,*l*_ is the quantum defect and the “+” superscript specifies that the respective value pertains to the ionic core. The energy contributions *E*_*i*_^+^ are given relative to the lowest rovibrational level of the cationic ground state. Sufficiently high principal quantum numbers *n* are assumed here, enabling a Hund’s case (d) description where the ionic core and the Rydberg electron barely interact, and their energy contributions can be separated. The Rydberg states are accordingly described by the core quantum numbers ν^+^, *N* ^+^ and the Rydberg electron quantum numbers *n*, *l*. From eq 1 it is evident that for *n* → ∞ a Rydberg series converges toward a rovibronic state of the cationic core.

For YbF, given the X^1^Σ^+^ state of the cation, and restricting ourselves to Rydberg series where the cationic core is not electronically excited, eq 1 takes on the form

2 $$E_{n,l,N^{+},\nu^{+}}=\mathrm{IE}+\omega_{e}^{+}\nu^{+}-\omega_{e}x_{e}^{+}\nu^{+}\left(\nu^{+}+1\right)+B^{+}N^{+}\left(N^{+}+1\right)-\frac{R_{\mathrm{YbF}}}{{\left(n-\delta_{n,l}\right)}^{2}}$$

where ω_*e*_^+^ is the vibrational frequency, ω_*e*_ *x*_*e*_^+^ is the anharmonicity, and ν^+^ and *N* ^+^ are the quantum numbers of vibration and total angular momentum (excluding nuclear and electronic spin), respectively. For a mass of ^174^YbF of 192.94 amu, *R*_YbF_ has the value 109,737.0 cm^–1^. As such, the Rydberg series is fully described by *E*_lim_(ν^+^, *N* ^+^) = *IE* + ω_*e*_^+^ ν^+^ – ω_*e*_ *x*_*e*_^+^ ν^+^(ν^+^ + 1) + *B* ^+^ *N* ^+^(*N* ^+^ + 1) and δ_*n*,*l*_. The quantum defect δ_*n*,*l*_ that quantifies the deviation of Rydberg states from ideal hydrogenic states due to the interaction of the Rydberg electron with the ionic core is known to be roughly constant at sufficiently high principal quantum number *n*.^10^

Table 1 shows the eight dominant Rydberg series that have been identified in the eight ν = 0 scans. A (roughly) *n*-independent quantum defect δ is assumed here, which is valid for the sufficiently high principal quantum numbers of the observed Rydberg states. Due to the methodology employed, which solely assigns effective quantum numbers n* ≡ *n* – δ to the Rydberg series members, the quantum defect can only be obtained in the form of a remainder value (i.e., modulo 1).

**Table 1:** Summary of all Identified Rydberg Series Converging to *N* ^+^, ν^+^ Rovibrational Levels of YbF^+^, along with their Determined Limit Energies and Quantum Defects (Mod 1)^a^

*J* in A^2^Π_1/2_	ν^+^	*N* ^+^	*E*_lim_ [cm^–1^]	*E*_lim_ – *E*_lim_^fit^ [cm^–1^]	mod_1_δ
0.5^–^	0	0	48706.68(21)^b^	0.11	0.99(1)
2.5^–^	0	2	48708.09(13)	–0.02	0.91(1)
4.5^–^	0	4	48711.74(20)	0.03	0.88(1)
5.5^+^	0	5	48714.00(25)	–0.29	0.82(2)
6.5^–^	0	6	48717.48(19)	0.11	0.87(1)
8.5^–^	0	8	48725.16(22)	0.07	0.86(2)
9.5^–^	0	10	48734.56(25)	–0.30	0.77(2)
10.5^+^	0	11	48740.68(23)	0.17	0.80(2)
0.5^–^	1	0	49305.36(15)	–0.07	0.79(1)
5.5^+^	1	5	49313.22(16)	0.13	0.97(1)
8.5^+^	1	9	49328.35(20)	–0.07	0.89(1)

a The residuals of the linear fit performed on the *E*_lim_(N^+^) values of same ν^+^ are also given.

b Uncertainties expressed as one standard deviation.

The assignment of the rotational quantum number, *N* ^+^, of the ionic core can be unambiguously made by comparing the eight limits of the different series to the expression for the rotational energy in the cation.

The spectroscopic constants *IE* and *B*_ν = 0_^+^ are extracted by a weighted least-square fit of *E*_lim_(ν^+^ = 0, *N* ^+^) to the expression for the rotational structure *E*_rot_ = *IE* + *B* ^+^ *N* ^+^(*N* ^+^ + 1) of the YbF^+^ X^1^Σ^+^ ground state. This leads to *IE* = 48,706.57(8) cm^–1^ and *B* ^+^ = 0.257(1) cm^–1^. It should be noted that the centrifugal distortion constant *D* = 4*B* ^3^ω_*e*_^–2^ is estimated to be in the range of 10^–7^ cm^–1^ for YbF^+^ and would have virtually no impact on the rotational energy levels of *N* ^+^ < 12. Hence, it was not included in the fit. The standard error of the fit, calculated from the residuals given in Table 1, is 0.07 cm^–1^.

The very same procedure was then utilized on the higher lying vibrationally excited Rydberg series shown in Figure 3, the limit values, quantum defects and rotational quantum numbers of which are also given in Table 1. The linear fit in this case led to a value of *E*_ν = 1_^+^ = 49,305.43(13) cm^–1^ and *B*_ν = 1_^+^ = 0.255(3) cm^–1^. Subtracting the *IE* value from the *E*_ν = 1_^+^ value yields the vibrational transition energy Δ*G*_1/2_ = ω_*e*_^+^ – 2ω_*e*_ *x*_*e*_^+^ = 598.86(15) cm^–1^.

**Figure 3:**
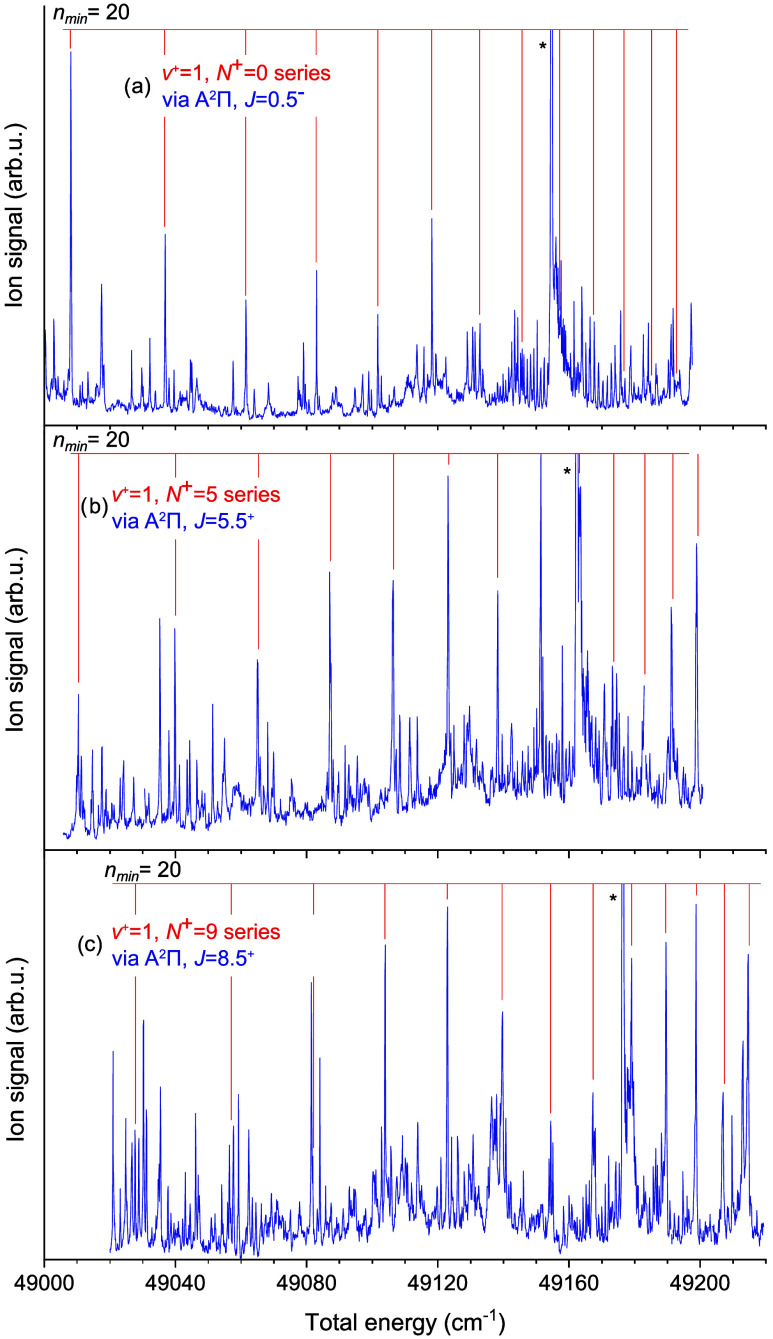
(1 + 1′)-REMPI spectra measured via three different rotational levels of A^2^Π_1/2_ along with the identified ν = 1 Rydberg series. The (lowest possible) *n* quantum number of a low-lying series member is given in the upper left corner of each spectrum.

The intense spectral feature marked by an asterisk in all three scans of Figure 3 is not due to states reached by means of the intended two-color (1 + 1′)-REMPI scheme via A^2^Π_1/2_, but due to one-color (1 + 1)-REMPI via the [31.05] state of YbF. This is evident from the fact that the feature appears at the same UV photon energy of ≈ 31,048.3 cm^–1^ in all three scans. The [31.05] mixed character state, named according to its term energy (in wavenumbers) divided by thousand, put in between square brackets, was previously identified and discussed by Persinger et al.^36^ and Popa et al.^37^ It is accessible from the X^2^Σ^+^ ground state, and the transition energies lie within the scanning range needed for the search for ν = 1 Rydberg series. The spectral feature marked by an asterisk is the bandhead of the R_1_ branch of the [31.05] ← X transition. It should be pointed out that this bandhead had been labeled as a Q branch in the work of Persinger et al., where its frequency was given as 31,052 cm^–1^.^36^ The correct labeling of the feature and its 3.7 cm^–1^ lower frequency found here is, however, confirmed by the more accurate, rotationally resolved study of Popa et al.^37^

Interestingly, when comparing Rydberg series of same *N* ^+^ but different ν, e.g., the ones from Figures 2(b) and 3(b), one notices that the ν = 1 series is not merely an up-shifted copy of the ν = 0 series, as evident from the different values of the quantum defects. This can be explained either by the influence of the core vibration on the Rydberg electron or by the two series pertaining to the same *N* ^+^ having different *l* values.

All of the determined spectroscopic constants for YbF and YbF^+^ are summarized and compared to values from earlier studies in Table 2. In particular, the *IE* value of YbF obtained here generally agrees with the one obtained by Persinger et al.^36^ using PFI-ZEKE, but is several cm^–1^ higher and much more accurate. The results for Δ*G*_1/2_ are also in good agreement.

**Table 2:** Summary of Spectroscopic Constants Derived from the Rydberg Series Analysis and Comparison to Other Studies^a^

	measurement				calculation
	^174^YbF^+^, this work	^174^YbF^+^,^36^	^174^YbF,^28^	^175^LuF,^53,54^	^174^YbF^+^,^35^
*E*_v=0_	48706.57(8)^b^	48703 ± 5^b^	0^b^	0^c^	48578^d^
*E*_v=1_	49305.43(13)^b^	49303.5 ± 5^b^	502.1772(37)^e^ ^,^ ^b^	606.71^c^ ^,^ ^e^	
Δ*G*_1/2_	598.86(15)	599.5 ± 1.3^e^	502.1772(37)^e^	606.71^e^	
*B*_ν=0_	0.257(1)		0.241416(22)	0.26686^e^	
*B*_ν=1_	0.255(3)		0.239881(21)	0.26530^e^	
*r*_ν=0_	1.957(4)		2.019139(90)^e^	1.9199^e^	
*r*_ν=1_	1.965(12)		2.025589(88)^e^	1.9256^e^	
ω_*e*_		604.9 ± 1.1	506.6674(94)^e^	611.79	
ω_*e*_ *x*_*e*_		2.7 ± 0.3	2.2451(43)^e^	2.54	

a Internuclear distances given in Å, all other values in cm^–1^. Uncertainties expressed as standard deviations.

b values relative to YbF, X^2^Σ^+^.

c values relative to LuF, X^1^Σ^+^.

d EOM-CCSD value in closest agreement to measurements.

e calculated from rovibrational constants explicitly given in reference.

From the rotational constants, the equilibrium bond length of YbF is seen to diminish by ca. 0.06 Å upon ionization, which is explained by the strong ionic character of the YbF bond^51^: The Coulomb interaction between the constituent Yb^+^ and F^–^ ions further increases upon removal of an electron from Yb^+^. In Table 2, a comparison is also made between the neutral luthetium monofluoride (LuF) molecule and YbF^+^, as both systems have closed shell singlet (^1^Σ^+^) ground states, namely Yb^2+^(4f^14^) F^–^ and Lu^+^(4f^14^6s^2^) F^–^.^52^ While the equilibrium bond lengths and the vibrational frequencies are indeed comparable, the LuF *r*_ν=0_ value is ca. 0.04 Å smaller than the YbF^+^ one, and the ω_*e*_ value of LuF is ca. 6.9 cm^–1^ larger (in spite of LuF being heavier), signifying that the LuF ionic bond is somewhat stronger.

While theoretical data to compare with the experimentally determined rovibrational parameters of the YbF cation is scarce, recently high level calculations on the *IE* of YbF have been performed.^35^ The EOM-CCSD value given in Table 2 differs from the measured value by approximately 130 cm^–1^, showcasing the state of the art of the relativistic quantum chemical calculations on lanthanide complexes.

The energy landscape of YbF in the first 40 cm^–1^ above the *IE* is densely packed, not only with the various ν = 0 Rydberg states observed here, but also by (at least) two electronically excited autoionizing core-hole states. These two states, labeled by their total energies and Ω quantum number as [48.72]­3/2 and [48.73]­1/2, have been fully rotationally characterized.^37^ Figure 4 shows how the rotational levels of the X^1^Σ^+^ state of YbF^+^ are positioned relative to the rotational levels of these two autoionizing states.

**Figure 4:**
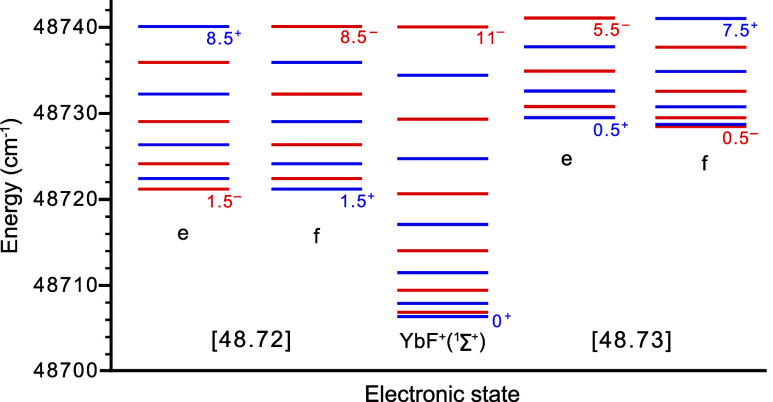
Autoionizing energy levels of YbF just above the field-free *IE*, along with the determined rotational levels of the ground state of YbF^+^. A multitude of Rydberg states converges toward each YbF^+^ level (not shown here). Within each stack of rotational levels, the total angular momentum quantum number (*N* for ^1^Σ^+^, otherwise *J*) increases in increments of one and alternates between positive (blue) and negative (red) parity. Lowest and highest *J* (or *N*) values are given for each stack.

### IR-PIRI

Infrared photoinduced Rydberg ionization has been performed for the *N* ^+^ = 4, n*­(=*n* – δ) = 36.12 Rydberg state, see Figure 5. This is a well-isolated state of sufficient transition strength prepared via *J* = 4.5^–^ in A^2^Π_1/2_ and lies at 48,627.63 cm^–1^, below the (field-free) *IE* of YbF. The scan range of the IR-FEL is selected to be around the vibrational transition frequency determined in the previous subsection. The IR-FEL causes nonresonant, direct photoionization of the molecule in the Rydberg state, as well as resonant vibrational excitation of the cationic core, followed by autoionization. The sought-after PIRI signal is therefore embedded in a background signal that is two to three times as large and that needs to be accounted for.

**Figure 5:**
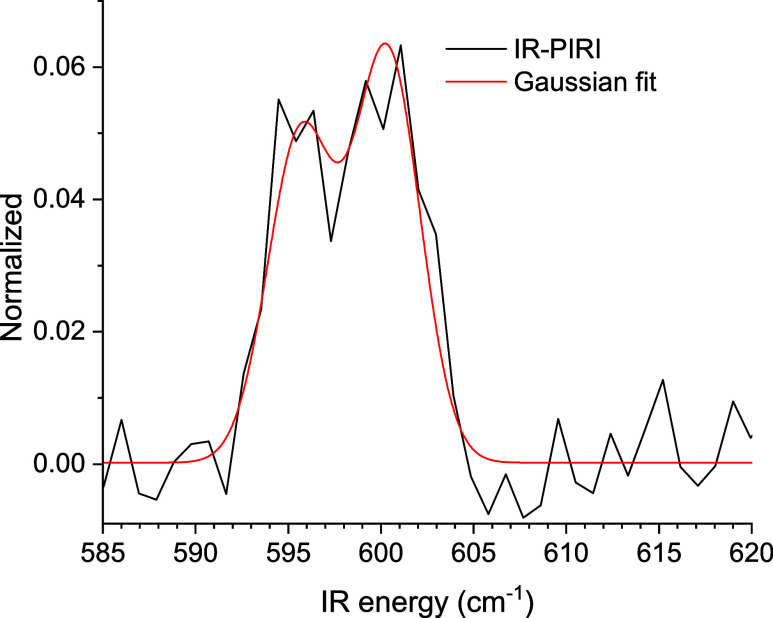
Averaged IR-PIRI spectrum in the region of the ν = 0 → 1 transition of YbF^+^.

Normalization of the ion signal is performed by dividing the IR laser-induced photoionization signal by the total signal, i.e., by the sum of photo- and field-induced signal, to account for short-term fluctuations in the number of YbF molecules in the specified Rydberg state. After normalization and averaging, the background is subtracted, leading to the PIRI spectrum displayed in Figure 5. Although somewhat noisy, it clearly shows a double peak structure.

Since the IR radiation essentially drives a vibrational transition of the YbF^+^ core (the Rydberg electron merely acting as a “tag”), both levels involved can be described in the same Hund’s case. The selection rules then require that the total angular momentum *N* ^+^ of the final state must either be identical to the one of the initial state, or differ from it by one, and that the parity must change. Hence, from *N* ^+′^ = 4 we are only able to transition into *N* ^+^ = 3 and *N* ^+^ = 5, which explains the two peaks observed in Figure 5.

The spacing Δ*E* between these peaks is expected to be 18*B* ^+^_ν = 1_ ≈ 4.5 cm^–1^. The vibrational transition energy Δ*G*_1/2_ lies between the central energies *E*_c_(*N* ^+^) of the two peaks, somewhat closer to the *N* ^+^ = 3 peak, as given by

3 $$\Delta G_{1/2}=E_{c}\left(5\right)-30B_{\nu =1}^{+}+20B_{\nu =0}^{+}=E_{c}\left(3\right)-12B_{\nu =1}^{+}+20B_{\nu =0}^{+}$$

With the rotational constants *B*_ν = 0_^+^ and *B*_ν = 1_^+^ obtained in the previous subsection, Δ*G*_1/2_ is hence determined to lie about 2.51 cm^–1^ below *E*_c_(*N* ^+^ = 5), or equivalently, 2.08 cm^–1^ above *E*_c_(*N* ^+^ = 3).

The Hönl-London factors that govern the intensity of the relevant transitions are given by^55^

4 $$S_{J\prime }^{P}=\frac{(J^{\prime }+\Lambda )\left(J^{\prime }-\Lambda \right)}{J^{\prime }}$$

5 $$S_{J\prime }^{R}=\frac{(J^{\prime }+1+\Lambda )\left(J^{\prime }+1-\Lambda \right)}{J^{\prime }+1}$$

where *J*′ is the total angular momentum quantum number of the initial level. For the ^1^Σ^+^ ground state of YbF^+^, eqs 4 and 5 take on the trivial forms *S*_*J*′_^*P*^ = *N*′ and *S*_*N*′_^*R*^ = *N*′ + 1 respectively. For *N*′ = 4 this leads to an expected intensity ratio of 0.8 between the allowed *P* and *R* transitions. The spectral structure of Figure 5 is fitted to two Gaussians with the distance between the central positions fixed to 4.59 cm^–1^ and the intensity ratio fixed to 0.8. Furthermore, the two widths of the Gaussians are taken to be equal. The two measured peaks follow the expected ratio of Hönl-London factors, indicating that the measurement was not performed in the saturation regime. The fitted width of the peaks is 3.5 ± 0.3 cm^–1^, and is mainly caused by the line width of the FEL and the finite lifetime of the final states. Since the FEL line width was only about 1.5 cm^–1^, a significant contribution must come from autoionization.

The fitted Δ*G*_1/2_ value (taking into account the accuracy of the FEL’s wavelength calibration) is 598(1) cm^–1^ and matches the values from Table 2, thereby confirming the assignment of the Rydberg spectra.

## Conclusions

Rotationally resolved (1 + 1’)-REMPI spectroscopy via the A^2^Π_1/2_ state of the ^174^YbF molecule has been performed, leading to the observation and identification of many Rydberg series converging to several rotational and vibrational levels of the ^174^YbF^+^ X^1^Σ^+^ ground state. The limit of each series, corresponding to the energy of a rovibrational level of the cation, has been extracted, and the respective quantum numbers ν^+^ and *N* ^+^ have been assigned. From the limit values of the nonvibrationally excited (ν = 0) Rydberg series an ionization energy of 48706.57(8) cm^–1^ (or 6.03884(1) eV) for YbF, along with the rotational constant *B*_ν = 0_^+^ of 0.257(1) cm^–1^ for the YbF^+^ cation in its X^1^Σ^+^ ground state have been determined. A rotational constant *B*_ν = 1_^+^ of 0.255(3) cm^–1^ and a term energy *E*_ν=1_ of 49305.43(13) cm^–1^ for the first vibrationally excited level of the X^1^Σ^+^ ground state of the YbF^+^ cation have also been obtained from an analogous analysis of vibrationally excited (ν = 1) Rydberg series, leading to a value for the vibrational transition energy Δ*G*_1/2_ of 598.86(15) cm^–1^. Additionally, the IR-PIRI technique has been applied to YbF in a rotationally state-selective manner, yielding rotationally resolved spectra that confirm the Δ*G*_1/2_ value obtained from the Rydberg series analysis. This makes YbF only the second heavy diatomic molecule successfully subjected to IR-PIRI,^56^ demonstrating the validity of the technique.

In future experiments, it might be worthwhile to look for the so far unobserved “4f hole” Rydberg states, potentially using a REMPI scheme via the [31.05] mixed character state described in^36,37^ , and coincidentally also observed in this work. Doing so could yield spectroscopic information on the low-lying 4f-hole states of YbF^+^, potentially leading to a better understanding of this unique, lanthanide-specific electronic configuration.
